# Analysis of Ionic-Exchange of Selected Elements between Novel Nano-Hydroxyapatite-Silica Added Glass Ionomer Cement and Natural Teeth

**DOI:** 10.3390/polym13203504

**Published:** 2021-10-12

**Authors:** Imran Alam Moheet, Norhayati Luddin, Ismail Ab Rahman, Sam’an Malik Masudi, Thirumulu Ponnuraj Kannan, Nik Rozainah Nik Abd Ghani

**Affiliations:** 1 Baqai Dental College, Baqai Medical University, Karachi 75340, Pakistan; ia_moheet@hotmail.com; 2 School of Dental Sciences, Universiti Sains Malaysia, Kubang Kerian 16150, Kelantan, Malaysia; arismail@usm.my (I.A.R.); kannan@usm.my (T.P.K.); rozainah@usm.my (N.R.N.A.G.); 3 Faculty of Dentistry, Lincoln University College, Petaling Jaya 47301, Selangor, Malaysia; masudi@usm.my

**Keywords:** ion-exchange, nano-hydroxyapatite, nano-silica, bonding, ionic-bonding, nano-HA-Si, SEM-EDX

## Abstract

One of the foremost missions in restorative dentistry is to discover a suitable material that can substitute lost and damaged tooth structure. To this date, most of the restorative materials utilized in dentistry are bio-inert. It is predicted that the addition of nano-HA-SiO_2_ to GIC matrix could produce a material with better ion-exchange between the restorative material and natural teeth. Therefore, the aim of the current study was to synthesize and investigate the transfer of specific elements (calcium, phosphorus, fluoride, silica, strontium, and alumina) between nano-hydroxyapatite-silica added GIC (nano-HA-SiO_2_-GIC) and human enamel and dentine. The novel nano-hydroxyapatite-silica (nano-HA-SiO_2_) was synthesized using one-pot sol-gel method and added to cGIC. Semi-quantitative energy dispersive X-ray (EDX) analysis was carried out to determine the elemental distribution of fluorine, silicon, phosphorus, calcium, strontium, and aluminum. Semi-quantitative energy dispersive X-ray (EDX) analysis was performed by collecting line-scans and dot-scans. The results of the current study seem to confirm the ionic exchange between nano-HA-SiO_2_-GIC and natural teeth, leading to the conclusion that increased remineralization may be possible with nano-HA-SiO_2_-GIC as compared to cGIC (Fuji IX).

## 1. Introduction

One of the foremost missions in restorative dentistry is to discover a suitable material that can substitute lost and damaged tooth structure. Therefore, a search for a material that possesses chemical, physical, and mechanical resemblance to natural tooth structures as well as minimizes the chances of further damage to the tooth. The continuous efforts lead to the discovery of various restorative materials among which glass ionomer cement stood out because of its various properties similar to natural tooth, even though they are not close to ideal material for tooth replacement [1,2,3,4,5,6,7]. To this date, most of the restorative materials utilized in dentistry are bio-inert. Historically, silicate cements exhibited fluoride ion release, which were taken up or absorbed into the surrounding tooth structures. Glass ionomer cement has shown similar property that has led to its major indication to be used as an anti-cariogenic restorative material [8,9,10].

The development of the atraumatic restorative technique (ART) in restorative dentistry has led to another interesting application of GIC in dentistry [11]. After the removal of caries and soft dentin up to the point where the patient feels pain, the ART method is utilized in restoring the cavity depending upon the capability of GIC to remineralize the remaining dentin [11]. Atraumatic restorative technique is currently being adapted by developing and developed countries for indirect pulp capping and there is scientific published material available for these materials exhibiting remineralization of partly demineralized dentin [12].

The bonding mechanism for self-cure cure glass ionomer cement to tooth structure was first postulated by Wilson et al. in 1983 [13]. Researchers have tried to verify this bond between tooth and restorative material through several mechanisms. Watson et al. incorporated fluorescent dyes in GIC to exhibit the possibility of ion-exchange between restorative material and tooth [14,15]. Ion mass spectrometry was used by Lin et al. to assess the bonding between tooth and restorative material [16]. While Ngo et al. demonstrated the presence of an interaction zone at the dentin-restorative interface using cryo-scanning electron microscopy. It was further suggested that ion-enriched layer is present at the interface which was resistant to acid etching [17]. These findings regarding ionic bonding were confirmed by Yoshida et al. With the help of X-ray photon spectrometry, the author reported ionic bonding between calcium and phosphorus within the tooth and carboxyl ions from the cement [18]. Utilizing electron probe microanalysis (EPMA) technique, Ngo et al. was successful in demonstrating the transference of fluoride and strontium from conventional GIC (cGIC) to demineralized dentin [19]. Similar work was performed by Knight et al. using EPMA demonstrating ion-exchange of selected elements between dentine and adhesive restorative materials [20]. These findings were confirmed by various authors [21,22,23]. In a more recent study on ion-exchange between cGIC and teeth (enamel and dentine) in relation with time was performed using energy dispersive X-ray spectrometer (EDS) [23]. The results re-confirm the exchange/migration of elements between cGIC and tooth in previous studies [23].

It is predicted that the addition of nano-HA-SiO_2_ to GIC matrix could produce a material with better ion-exchange between the restorative material and natural teeth. Therefore, a detailed evaluation of elemental exchange between the nano-HA-silica added GIC and natural teeth is crucial before any recommendations can be made. To the best of our knowledge, the ionic-exchange property of nano-HA-silica-GIC is yet to be determined. Therefore, the aim of the current study was to investigate the transfer of specific elements (calcium, phosphorus, fluoride, silica, strontium, and alumina) between nano-hydroxyapatite-silica added GIC and human enamel and dentine and compare it with conventional GIC (Fuji IX).

## 2. Materials and Methods

This in vitro study was granted approval for implementation by Jawatankuasa Etika Penyelidikan Manusia Universiti Sains Malaysia (JEPeM-USM) bearing the protocol code USM/JEPeM/18080371.

### 2.1. Materials

Commercialized glass ionomer cement (Fuji IX GP, GC International, Tokyo, Japan) in powder and liquid states were used in this study. The rest of the chemicals used were of analytical grade. The chemical used in the current study were calcium hydroxide (<98%, RM Chemicals, Bhopal, India), phosphoric acid (<99%, Sigma-Aldrich, Darmstadt, Germany), tetraethyl orthosilicate (TEOS, 99%, Fluka, Seelze, Germany), ethanol (99%, Systerm, Selangor, Malaysia), ammonia (99%, Sigma-Aldrich, Darmstadt, Germany) and total ionic strength buffer III (TISAB, Sigma-Aldrich, Darmstadt, Germany).

### 2.2. Synthesis of Nano-Hydroxyapatite-Silica Powder

Nano-hydroxyapatite-silica powder was synthesized by a one-pot sol-gel technique [24,25,26,27,28]. A total of 7.408 g of calcium hydroxide was dissolved in 100 mL of distilled water. This suspension was mixed with a magnetic stirrer for 30 min. 4.104 mL of phosphoric acid was added drop-wise to calcium hydroxide suspension [24,25,26,27,28]. This suspension was stirred for 48 h. Liquid ammonia was used to maintain the pH of the suspension between 11–12. A quantity of 20 mL TEOS was dissolved in 10 mL of absolute ethanol and was added drop-wise to calcium hydroxide suspension after 12 h. After 48 h, the sol produced was centrifuged (Eppendorf Centrifuge 5804, Darmstadt, Germany) followed by freeze-drying (ScanVac CoolSafe, Lillerød, Denmark) and calcined (WiseTherm, Lilienthal, Germany) at 600 °C. The calcined powder was ground manually using a mortar and pestle for 10 min [24,25,26,27,28].

### 2.3. Preparation of Nano-Hydroxyapatite-Silica Added GIC Powder

Glass ionomer cement was hand mixed following the manufacturers’ instruction by using 1:1 powder/liquid ratio. Nano-HA-SiO_2_ powder was weighed and added to cGIC powder at a percentage by weight of 10%. The powder mixture of nano-HA-SiO_2_ and cGIC was manually mixed by mortar and pestle for 10 min [24,25,26,27,28]. Nano-hydroxyapatite-silica-GIC (nano-HA-SiO_2_-GIC) samples were prepared following the same powder/liquid proportions.

### 2.4. Preparation of Samples for Ionic-Exchange Evaluation

Ten permanent human premolars extracted for orthodontic and periodontal reasons were used for this study. The crowns of the teeth were separated from the roots using a high-speed dental hand piece with water cooling at the level of the cemento-enamel junction. Remnants of the pulp tissue was discarded. Coronal segments of the teeth were thoroughly ultra-sonicated and polished with pumice and polishing toothpaste. Cervical class V cavities were prepared on the buccal surface of each tooth using a regular grit fissure diamond bur and high-speed dental hand piece with water cooling. The dimensions of the cavities were: mesio-distal width 2.5 ± 0.3 mm, occluso-gingival width 1.5 ± 0.3 mm, depth 1.5 ± 0.3 mm [28]. The cavity measurements were checked and confirmed with a digital gauge. The gingival edge of the preparation was above the cemento-enamel junction (in enamel). The teeth were randomly divided into two groups and restored with conventional GIC and nano-HaSiO_2_-GIC. Samples were stored in de-ionized water at 37 ± 1 °C for 3, 7, 15 and 30 days.

**Figure 1:**
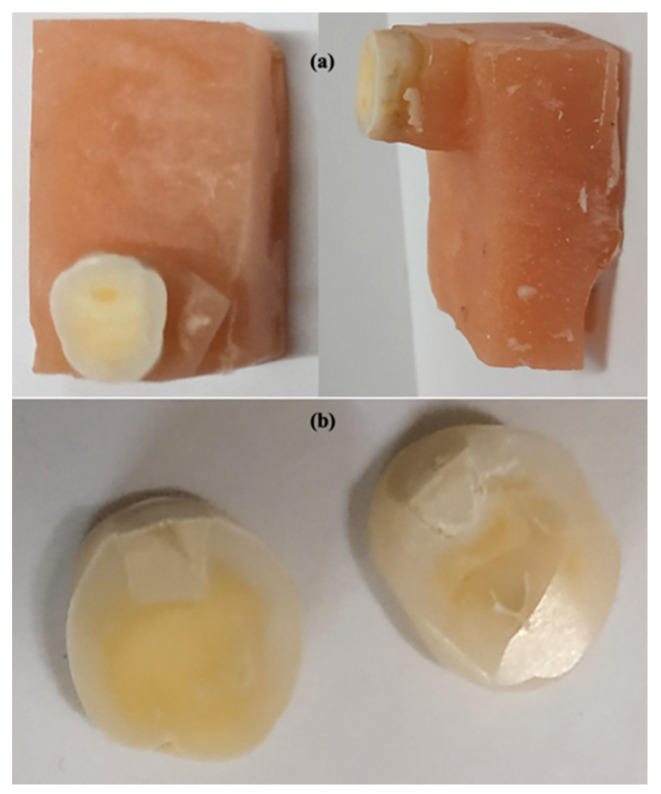
Cross-section of teeth for ion-exchange experimentation (**a**) teeth in acrylic for sectioning and (**b**) horizontal section of teeth for ion-exchange experimentation.

Following storage in de-ionized water, the teeth were sectioned horizontally in bucco-lingual direction (Figure 1a,b). The cut surface of the samples was ground flat with water-cooled carborundum discs (320, 600, and 1200 grits of Al_2_O_3_ papers: Buehler, Lake Bluff, IL, USA), and polished with diamond polishing paper (Polishing Paper 1 Micron 8000 Grit).

**Figure 2:**
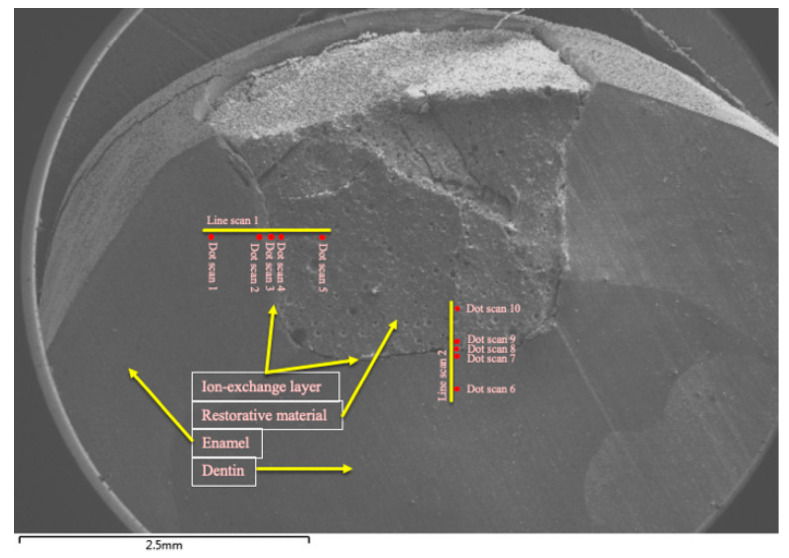
Scan zones for analysis of ion-concentration.

Semi-quantitative energy dispersive X-ray (EDX) analysis was carried out to determine the elemental distribution of fluorine, silicon, phosphorus, calcium, strontium, and aluminum. EDX analysis was performed by collecting line-scans and dot-scans. These scans were made along a line between restorative material (cGIC and nano-HA-SiO2-GIC) and enamel/dentine. A total of 5 dot-scans for quantitative analysis were performed and 1 line-scan was performed for qualitative assessment. A total of 10 dot-scan locations were selected for each specimen. In total, there were 5 dot-scans for enamel-restoration ion-exchange analysis and 5 dot-scans for dentine-restoration ion-exchange analysis (Figure 2). One dot-scan each was conducted starting at a distance of 0.5 and 0.1 mm within the restoration adjacent to the tooth restorative interface. One dot-scan was performed at the tooth restorative interface referred as to Ion-exchange Layer (IEL) and one dot-scan each was performed at distance of 0.1 and 0.5 mm into the enamel and dentine covering a total distance of 1 mm for all scans combined (Figure 2). One line-scan was conducted on the same location and of the same length for qualitative analysis. Measurements were expressed as a relative percentage weight of the identified element as part of the total weight of the sample where the measurement was taken [23].

### 2.5. Statistics

All the data were analyzed using SPSS version 23 (IBM Corp., New York, NY, USA). Analysis of variance (ANOVA), with post-hoc Tukey test was used to determine the intra-group mean differences for nano-HA-SiO_2_-GIC and cGIC for ionic-exchange recorded at different distance as well as for different time intervals. Independent two-tailed *t*-test was used to find out mean differences between cGIC and nano-HA-SiO_2_-GIC for ionic-exchange at 95% confidence interval. A value of α = 0.05 was considered statistically significant.

## 3. Results

The mean and standard deviation of ion-concentration for selected elements in experimental samples recorded at different time intervals are given Table 1, Table 2, Table 3 and Table 4. The complete data set of the current study is provided in a supplementary file.

**Figure 3:**
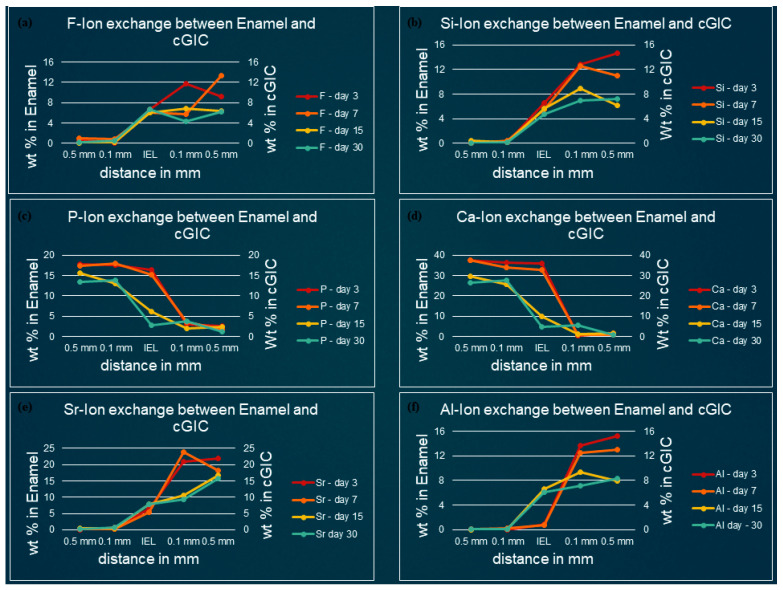
Ion-exchange between cGIC and enamel (**a**) fluorine, (**b**) silicon, (**c**) phosphorus, (**d**) calcium, (**e**) strontium, and (**f**) aluminum.

**Figure 4:**
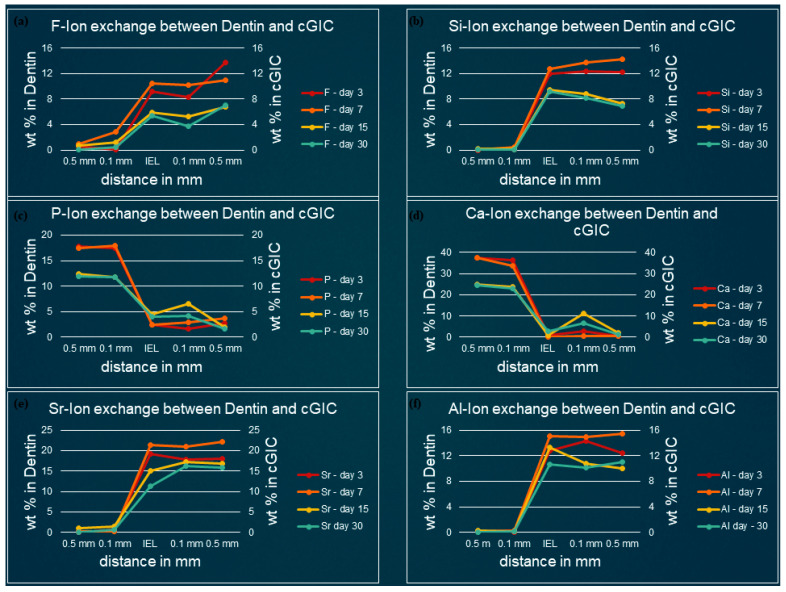
Ion-exchange between cGIC and dentin (**a**) fluorine, (**b**) silicon, (**c**) phosphorus, (**d**) calcium, (**e**) strontium, and (**f**) aluminum.

**Figure 5:**
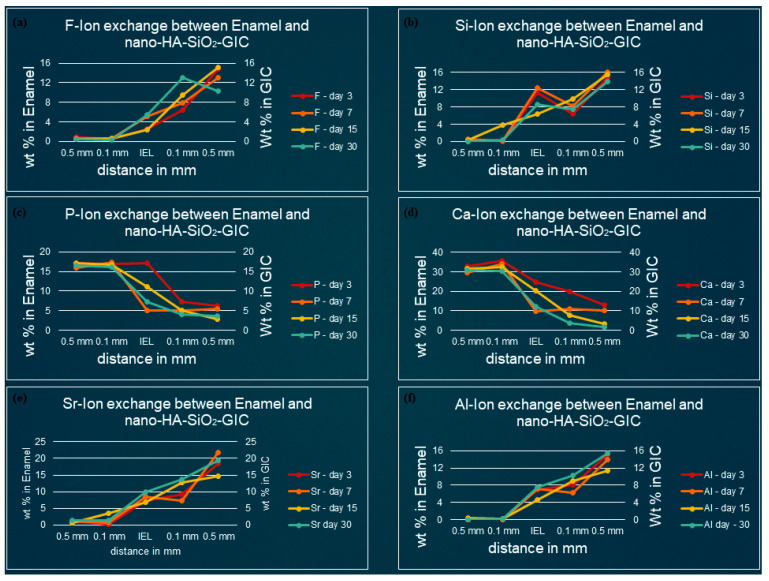
Ion-exchange between nano-HA-SiO_2_-GIC and enamel (**a**) fluorine, (**b**) silicon, (**c**) phosphorus, (**d**) calcium, (**e**) strontium, and (**f**) aluminum.

**Figure 6:**
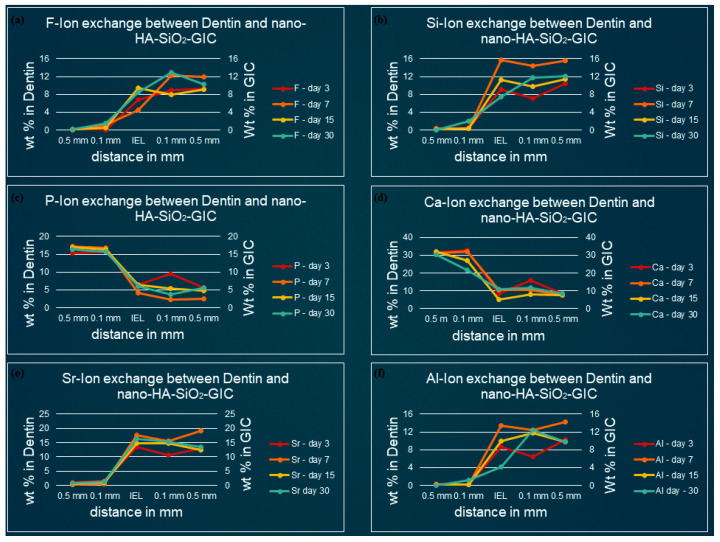
Ion-exchanged between nano-HA-SiO_2_-GIC and dentin (**a**) fluorine, (**b**) silicon, (**c**) phosphorus, (**d**) calcium, (**e**) strontium, and (**f**) aluminum.

Figure 3, Figure 4, Figure 5 and Figure 6 show the graphical representation of weight percentage (wt.%) of the element’s fluoride, silicon, phosphorus, calcium, strontium, and aluminum detected at various depth in the restorations and in the adjacent enamel and subjacent dentine at Day 3, 7, 15 and 30. Fluoride, silicon, strontium, and aluminum ions all displayed a similar trend (Figure 3, Figure 4, Figure 5 and Figure 6). They were distributed in greater concentrations towards the cGIC and nano-HA-SiO_2_-GIC with lower ionic concentration at ion exchange layer (IEL) and an even lesser amount was detected at adjacent enamel and subjacent dentine surface. Calcium and phosphorus ions, on the contrary, had higher concentrations towards the enamel and dentine and displayed a downward concentration trend for all samples towards IEL and an even lower concentration adjacent to cGIC and nano-HA-SiO_2_-GIC (Figure 3, Figure 4, Figure 5 and Figure 6).

### 3.1. Ion-Concentration of Various Selected Elements in Enamel/Dentine and cGIC at Various Time Intervals

**Fluoride:** Similar pattern of fluoride ion concentrations were detected in all cGIC samples at various time intervals (Figure 3a and Figure 4a). Fluoride ion concentration between 10 and 13 wt.% was recorded for day 3 and day 7 cGIC samples, whereas slightly less fluoride ion concentration (6 and 7 wt.%) was observed for day 15 and day 30 cGIC samples. The level of fluoride ion concentration slightly decreased as it approached IEL for all samples. There was some evidence of the presence of fluoride ion in the adjacent enamel and subjacent dentine surface. Subjacent dentine surface exhibited slightly greater amount of fluoride ion presence as compared to adjacent enamel surface for cGIC samples at day 15 and day 30 (Figure 3a and Figure 4a).

**Silicon:** The distribution of silicon in the body of cGIC was reported at a concentration of 11–15 wt.% for day 3 and day 7 cGIC samples, whereas 6–8 wt.% was observed for day 15 and day 30 cGIC samples. Silicon ion concentration for all samples dropped significantly at IEL adjacent to enamel, whereas silicon ion concentration adjacent to dentine was maintained at IEL for day 3 and day 7 cGIC samples and there was a slight increase in silicon concentration for day 15 and day 30 cGIC samples followed by a sharp drop in silicon ion concentration. Only trace amounts were detected in the adjacent enamel and subjacent dentine surfaces (Figure 3b and Figure 4b).

**Calcium and Phosphorus:** The distribution of calcium and phosphorus in the body of the enamel and dentine followed a similar pattern to each other for various cGIC samples. Calcium was approximately 38 wt.% while phosphorus was approximately 18 wt.% for day 3 and day 7 cGIC samples. These levels were maintained at IEL adjacent to enamel surface for day 3 and day 7 cGIC samples. On the contrary, these levels dropped significantly at IEL adjacent to enamel surface for day 15 and day 30 cGIC samples (Figure 3c,d), with the presence of a small concentration of calcium and phosphorus in the adjacent enamel surface. In the meantime, the concentration for calcium and phosphorus ion adjacent to the dentine surface for all samples dropped significantly at IEL. A slight peak of concentration for calcium and phosphorus ions were detected in the subjacent dentine (Figure 3c,d and Figure 4c,d).

**Strontium and aluminum:** Strontium ion was detected in the body of cGIC at a concentration of 18–22 wt.% for day 3 and day 7 samples, while a lower concentration of strontium was recorded (15–17 wt.%) for day 15 and day 30 cGIC samples. There was a sharp decline in concentration of strontium ion at IEL adjacent to enamel surface for all cGIC samples (Figure 3e). Higher levels of strontium ion were observed at IEL adjacent to dentine for day 3 and day 7 cGIC samples (Figure 4e). Day 15 and day 30 cGIC samples had a slightly lower level of strontium ion at IEL adjacent to dentine as compared to the body of restoration. Trace amounts of strontium were observed in the adjacent enamel and subjacent dentine surfaces. The level of aluminum ion observed were lower (13–16 wt.% for day 3 and day 7 and 7–10 wt.% for day 15 and day 30) as compared to strontium. However, it followed the same general distribution pattern as the strontium ion (Figure 3e,f and Figure 4e,f).

### 3.2. Ion-Concentration of Various Selected Elements in Enamel/Dentine and Nano-HA-SiO_2_-GIC at Various Time Intervals

**Fluoride:** For nano-HA-SiO_2_-GIC samples the fluoride ion distribution presented with a similar trend for samples. The fluoride ion concentration was between 10–15 wt.% in the body of the restoration. A lower concentration of fluoride ion (2–6 wt.%) was recorded at IEL adjacent to enamel, while slightly higher values (6–10 wt.%) were observed at IEL next to dentine surface. These levels dropped significantly, and negligible amounts of fluoride ion were detected in the adjacent enamel and subjacent dentine (Figure 5a and Figure 6a).

**Silicon:** The distribution of silicon ion was recorded between 10–15 wt.% for all nano-HA-SiO_2_-GIC samples. These levels dropped slightly at IEL and a concentration of 6–15 wt.% was observed at IEL adjacent to the enamel and dentine surface (Figure 5b and Figure 6b). These levels further decreased significantly for all nano-HA-SiO_2_-GIC samples, and trace amount of silicon was detected in the adjacent enamel and subjacent dentine surfaces (Figure 5b and Figure 6b).

**Calcium and phosphorus:** The distribution of calcium and phosphorus in the body of the enamel and dentine followed a similar pattern to each other for various nano-HA-SiO_2_-GIC samples (Figure 5c,d and Figure 6c,d). Calcium was approximately 32 wt.% while phosphorus was approximately 18 wt.% for all nano-HA-SiO_2_-GIC samples. There was a general decline in concentration for calcium and phosphorus ions at IEL. The concentration for calcium and phosphorus ions recorded at IEL adjacent to enamel were higher than the values recorded at IEL next to dentine for various nano-HA-SiO_2_-GIC samples. A significant amount of calcium and phosphorus was detected at the adjacent enamel and dentine surface.

**Strontium and aluminum:** Strontium ion was detected in the body of nano-HA-SiO_2_-GIC at a concentration of 18–22 wt.% for all samples, while a slightly lower concentration of aluminum ion was recorded (9–15 wt.%) for various nano-HA-SiO_2_-GIC samples. There was a sharp decline in concentration of strontium and aluminum ions at IEL adjacent to enamel surface for all cGIC samples (Figure 5e,f and Figure 6e,f). Higher levels of strontium and aluminum ions were observed at IEL adjacent to dentine for all nano-HA-SiO_2_-GIC samples. Detectable levels of strontium and aluminum ions were present in the adjacent enamel and subjacent dentine surfaces for all nano-HA-SiO_2_-GIC samples.

## 4. Discussion

Ionic exchange between glass ionomer cement and tooth structure requires an aqueous environment in order for ionic exchange to occur [29,30]. Calcium and phosphate ions are dispersed from hydroxyapatite into the cement. Phosphate ions buffer the polyalkenoic acid from the cement within the tooth. Ions are also dispersed from the unset cement into the adjacent tooth structure. As a result, an intermediate layer is formed between GIC and the tooth structure, commonly referred to as the “ion-exchange layer (IEL)” [31]. When viewed through an SEM, this ion-exchange layer appears to be several micro-meters wide (32,33). However, in the current study the extent of ionic exchange observed using SEM-EDX between restorative material and natural tooth structure was till 0.5 mm either side of the tooth-GIC interface that was well beyond the normal extent of IEL (Figure 2).

The permeation of elements from restorative materials to dental tissues is reported in several studies [19,32,33,34,35,36,37,38]. In vitro studies carried out by Hotta et al. (2001) and Extercate et al. (2005) on bovine teeth revealed an elevated fluoride ion level in the dentine adjacent to glass ionomer fillings. Tveit et al. (1980) in an in vitro study evaluated the absorption of fluoride ion released from fluoride-enriched amalgamates [39]. The author reported that fluoride ion was absorbed better by dentine than enamel. This finding seems to be consistent with the ionic exchange for the current study. According to Tveit et al. (1981), such findings are advocated as they is associated with dentine structure, whereby it has higher content of the organic part and water and lower crystallization rate. Murai et al. (1993), in an in vivo study confirmed elevated fluoride ion levels in the dentine adjacent to Vitrabond^®^ fillings. Similar to the results reported in the current study, Murai et al. (1993) also found that the highest concentration of fluoride ion directly adjacent or subjacent to the restorative material, which decreased towards the farther pulpal surface that indicates free penetration of fluoride ion inside the dentine. However, conflicting evidence was provided by Massara et al. (2002), who found no presence of fluoride ions in the dentine under the Fuji IX restorations. Wesenberg and Hals (1980), assessed the in vitro effect of glass ionomer cement (ASPA) on the mineral composition of enamel and dentine of human teeth [40]. The authors determined the content of fluoride ion in tooth structure, 1–3 months after the restoration was placed. The authors reported an increased level of fluoride ions in the tooth structure adjacent to the restoration. The results reported in the current study are in consistence with the results reported by Wessenberg and Hals (1980) and Marczuk-Kolada (2006). In this case, the current study reported an increase fluoride and aluminum ion levels in the tissues adjacent to nano-HA-SiO_2_-GIC restorations, the levels being statistically higher in dentine than in enamel. The data suggests that although more fluoride ions are being released into the environment, there is a limit to the amount of fluoride that is capable of being deposited into enamel and dentine from the restorative material.

The strontium and aluminum ions in the adjacent enamel and subjacent dentine may have been derived from the inorganic filler fraction of the nano-HA-SiO_2_-GIC. Studies using self-etching primers for dentine bonding on extracted human teeth have shown extensive penetration of resin tags into the dentine tubules [19]. In the current study, it is not clear whether strontium and aluminum ions followed such a pathway, or if they were present inside the tooth structure as a result of the diffusion process. The current study supports the concept of a chemical bond between tooth structure and nano-HA-SiO_2_-GIC and furthers enhances the confidence of the future clinical application of this material.

In agreement to Marczuk-Kolada (2006), an increase in levels of Ca and P ions was reported. However, there was no significant differences in the levels of Ca and P ions as well as in the Ca/P ratio in both enamel and dentine. There was evidence of a slight spike occurring in both the calcium and phosphorus ion levels close to the restorative interface that indicates calcium and phosphorus ions may have become incorporated from the dentine and enamel into the nano-HA-SiO_2_-GIC. However, some of the results reported in the current study were contradicted to Wessenberg and Hals (1980). In contrast to their findings, a rise in the concentration of silicon ion in the dentine adjacent to the filling as compared to enamel was observed. In the current study, which is in agreement with the findings reported by Marczuk-kolada (2006). Knychalska-Karwan and Pawlicki (1999), Pawlicki and Knychalska-Karwan (1994) and Szczepańska (1999), some findings were reported of calcium and phosphorus ions in the enamel of the deciduous teeth [41,42,43]. The authors reported lower weight percent values of these elements than those obtained in our study. However, it should be remembered that the content of these elements in the hard dental tissues is inter-changeable [44]. According to Pawlicki and Knychalska-Karwan (1994), the concentration of ion in the deciduous teeth undergoes variations with the progression of tooth resorption. In our analysis, the levels of calcium and phosphorus ions in the dentine adjacent to the restorative material were significantly lower than those at 1 mm distant. Nevertheless, Wessenberg and Hals (1980) have obtained contradictory findings with regards to calcium and phosphorus ions. However, their experiment was carried out on permanent and not deciduous teeth. Glass ionomer cement (ASPA) restorations were placed in experimentally formed cavities in the intact dental tissue. Massara et al. (2002) experimented on GIC (Fuji IX) using ART, reported elevated levels of calcium ions in the dentine close to the restorative material that is in agreement with the results reported in the current study. The Ca/P ratio obtained in the current study is almost similar with those presented by other authors assessing dentine composition after chemo-mechanical treatment of caries [45,46]. The lower concentrations of calcium and phosphorus in the dentine directly under the filling as compared to the distant sites may suggest the presence of partly demineralized dentine on the cavity floor. This suggestion was also made by Angker et al. (2004) [47].

The significant increase in weight percent values of fluorine, aluminum, calcium, phosphate, strontium, and silicon in the dentine adjacent to nano-HA-SiO_2_-GIC cement may indicate the passing of these elements from the restoration to the tooth. As reported by some of the authors certain elements such as aluminum, fluorine, strontium and silicon are likely to replace calcium and perhaps phosphorus in apatite’s [48,49,50]. The ions released from both the experimental cement and the tooth structure will combine to buffer the low pH until such time, the pH rises to a level where the ionic activity ceases. During this period of activity, both fluorine and strontium ions are available to undertake apatitic activity. This apatitic activity is more concentrated in calcium deprived regions of dentine, where strontium ions replace the missing calcium ions. It is suggested that this occurs through a diffusion process driven partly by the concentration gradient, which exists between the glass ionomer and the dentine with respect to these two elements [19]. As both strontium and fluorine are apatite-forming elements, they react with the demineralized dentine. This process is purely controlled by diffusion; therefore, one would expect to see the level of strontium and fluorine to be highest at the interface and lowest deep into the sound dentine [19]. This fact is evident in the current study where we can see increase wt.% of strontium and fluoride ions at IEL and trace amounts were recorded as we go deeper towards the sound tooth structure.

## 5. Conclusions

In conclusion, the results of the current study seem to confirm the ionic exchange between nano-HA-SiO_2_-GIC and natural teeth, leading to an assumption that increased remineralization may be possible with nano-HA-SiO_2_-GIC as compared to cGIC (Fuji IX) since it involves elements permeating from the restorative material into the tooth. Thus, the hypothesis for the current study that the addition of nano-hydroxyapatite-silica powder to conventional GIC enhances the chemical properties of GIC in terms of ionic-exchange is accepted.

## Figures and Tables

**Table 1:** Mean and standard deviation of various selected ions in enamel and dentine at day 3 expressed in wt.%.

	Experimental Groups	Enamel			Dentine		
		0.5 mm	0.1 mm	IEL-Enamel	IEL-Dentine	0.1 mm	0.5 mm
Fluoride	cGIC	0.775 ± 0.103 ^3,4^	0.125 ± 0.054 ^3,4^	6.963 ± 0.677 ^1,2,4,5,6^	8.701 ± 0.69 ^1,2,3,5,6^	0.114 ± 0.038 ^3,4^	0.493 ± 0.067 ^3,4^
	Nano-HA-SiO_2_-GIC	1.03 ± 0.311 ^2,3,4,5,6^	0.389 ± 0.111 ^1,3,4,5,6^	2.622 ± 0.027 ^1,2,4,5,6^	6.838 ± 0.021 ^1,2,3,5,6^	0.053 ± 0.017 ^1,2,3,4^	0.101 ± 0.014 ^1,2,3,4^
	*p*-value (*t*-test)	0.000 **	0.001 **	0.000 **	0.000 **	0.011 *	0.000 **
Silica	cGIC	0.002 ± 0.004 ^3,4^	0.138 ± 0.046 ^3,4^	6.373 ± 0.68 ^1,2,5,6^	6.99 ± 0.596 ^1,2,5,6^	0.162 ± 0.039 ^3,4^	0.004 ± 0.005 ^3,4^
	Nano-HA-SiO_2_-GIC	0.685 ± 0.18 ^3,4^	0.271 ± 0.07 ^3,4^	11.95 ± 1.539 ^1,2,4,5,6^	8.969 ± 0.259 ^1,2,3,5,6^	0.56 ± 0.172 ^3,4^	0.239 ± 0.046 ^3,4^
	*p*-value (*t*-test)	0.000 **	0.008 **	0.000 **	0.000 **	0.001 **	0.000 **
Phosphorus	cGIC	17.82 ± 0.131 ^3,4^	17.526 ± 0.03 ^3,4^	16.446 ± 0.285 ^1,2,4,5,6^	2.679 ± 0.397 ^1,2,3,5,6^	17.53 ± 0.043 ^3,4^	17.859 ± 0.158 ^3,4^
	Nano-HA-SiO_2_-GIC	16.876 ± 0.294 ^4^	16.954 ± 0.056 ^4^	17.057 ± 0.015 ^4^	6.426 ± 0.019 ^1,2,3,5,6^	15.323 ± 3.889 ^4^	15.927 ± 1.759 ^4^
	*p*-value (*t*-test)	0.000 **	0.000 **	0.001 **	0.000 **	0.24	0.04 *
Calcium	cGIC	37.385 ± 0.195 ^4^	36.269 ± 0.022 ^4^	36.248 ± 0.56 ^4^	0.649 ± 0.447 ^1,2,3,5,6^	37.067 ± 4.142 ^4^	36.399 ± 2.416 ^4^
	Nano-HA-SiO_2_-GIC	32.973 ± 0.127 ^3,4^	35.561 ± 0.071 ^3,4,5,6^	24.784 ± 0.043 ^1,2,4,5,6^	8.267 ± 0.033 ^1,2,3,5,6^	30.722 ± 1.603 ^2,3,4^	32.043 ± 2.882 ^2,3,4^
	*p*-value (*t*-test)	0.000 **	0.000 **	0.000 **	0.000 **	0.013	0.032
Strontium	cGIC	0.002 ± 0.004 ^3,4^	0.7 ± 0.017 ^3,4^	6.398 ± 0.393 ^1,2,4,5,6^	19.692 ± 1.516 ^1,2,3,5,6^	0.681 ± 0.051 ^3,4^	0.002 ± 0.004 ^3,4^
	Nano-HA-SiO_2_-GIC	1.191 ± 0.231 ^2,3,4^	0.125 ± 0.06 ^1,3,4,5,6^	7.408 ± 0.025 ^1,2,4,5,6^	12.768 ± 1.005 ^1,2,3,5,6^	1.558 ± 0.082 ^2,3,4^	1.124 ± 0.184 ^2,3,4^
	*p*-value (*t*-test)	0.000 **	0.000 **	0.000 **	0.000 **	0.000 **	0.000 **
Aluminum	cGIC	0.093 ± 0.009 ^4^	0.002 ± 0.004 ^4^	0.998 ± 0.349 ^4^	11.87 ± 2.023 ^1,2,3,5,6^	0.002 ± 0.004 ^4^	0.098 ± 0.002 ^4^
	Nano-HA-SiO_2_-GIC	0.139 ± 0.072 ^3,4,6^	0.168 ± 0.12 ^3,4,6^	7.069 ± 0.016 ^1,2,4,5,6^	8.564 ± 0.032 ^1,2,3,5,6^	0.237 ± 0.105 ^3,4^	0.393 ± 0.099 ^1,2,3,4^
	*p*-value (*t*-test)	0.186	0.015 *	0.000 **	0.006 **	0.001 **	0.000 **

* Indicates significant difference between cGIC and nano-HA-SiO_2_-GIC (*p* ≤ 0.05). ** Indicates highly significant difference between cGIC and nano-HA-SiO_2_-GIC (*p* ≤ 0.05). In each row, different superscript numbers indicate intra group significant differences between each material (*p* < 0.05).

**Table 2:** Mean and standard deviation of various selected ions in enamel and dentine at day 7 expressed in wt.%.

	Experimental Groups	Enamel			Dentine		
		0.5 mm	0.1 mm	IEL-Enamel	IEL-Dentine	0.1 mm	0.5 mm
Fluoride	cGIC	1.096 ± 0.149 ^3,4,5^	0.917 ± 0.339 ^3,4,5^	4.678 ± 1.528 ^1,2,4,5,6^	10.05 ± 1.246 ^1,2,3,5,6^	3.026 ± 0.303 ^1,2,3,4,6^	0.937 ± 0.184 ^3,4,5^
	Nano-HA-SiO_2_-GIC	0.677 ± 0.175 ^3,4^	0.63 ± 0.09 ^3,4^	4.124 ± 2.394 ^1,2,5,6^	4.955 ± 1.066 ^1,2,5,6^	1.196 ± 0.064 ^3,4^	0.075 ± 0.036 ^3,4^
	*p*-value (*t*-test)	0.004 **	0.108	0.674	0.000 **	0.000 **	0.000 **
Silica	cGIC	0.082 ± 0.011 ^3,4^	0.633 ± 0.209 ^3,4^	5.842 ± 1.75 ^1,2,4,5,6^	12.46 ± 1.548 ^1,2,3,5,6^	0.453 ± 0.217 ^3,4^	0.073 ± 0.009 ^3,4^
	Nano-HA-SiO_2_-GIC	0.685 ± 0.18 ^2,4,5,6^	0.271 ± 0.07 ^1,3,4^	11.95 ± 1.539 ^2,4,5,6^	17.53 ± 1.009 ^1,2,3,5,6^	0.314 ± 0.081 ^1,3,4,^	0.296 ± 0.045 ^1,3,4^
	*p*-value (*t*-test)	0.000 **	0.006 **	0.000 **	0.000 **	0.216	0.000 **
Phosphorus	cGIC	17.606 ± 0.153 ^3,4^	17.936 ± 0.466 ^3,4^	14.133 ± 1.499 ^1,2,4,5,6^	3.447 ± 2.222 ^1,2,3,5,6^	17.828 ± 0.205 ^3,4^	17.458 ± 0.175 ^3,4^
	Nano-HA-SiO_2_-GIC	15.977 ± 0.153 ^3,4^	17.41 ± 0.081 ^3,4^	6.3493 ± 1.342 ^1,2,5,6^	4.582 ± 1.492 ^1,2,5,6^	16.863 ± 1.24 ^3,4^	16.576 ± 1.816 ^3,4^
	*p*-value (*t*-test)	0	0.038 *	0	0.371	0.125	0.311
Calcium	cGIC	37.499 ± 0.266 ^2,3,4,6^	33.845 ± 0.241 ^1,5,6^	31.414 ± 1.189 ^1,5,6^	0.136 ± 0.01 ^1,5,6^	33.864 ± 1.628 ^2,3,4,5,6^	36.874 ± 2.611 ^1,2,3,4,5^
	Nano-HA-SiO_2_-GIC	29.707 ± 0.12 ^2,3,4^	34.022 ± 0.05 ^1,3,4^	11.277 ± 3.066 ^1,2,5,6^	11.15 ± 1.23 ^1,2,5,6^	31.019 ± 2.523 ^3,4^	30.865 ± 0.852 ^3,4^
	*p*-value (*t*-test)	0.000 **	0.146	0.000 **	0.000 **	0.067	0.001 **
Strontium	cGIC	0.415 ± 0.044 ^3,4^	0.265 ± 0.101 ^3,4^	6.016 ± 1.803 ^1,2,4,5,6^	20.208 ± 3.133 ^1,2,3,5,6^	0.623 ± 0.146 ^3,4^	0.297 ± 0.151 ^3,4^
	Nano-HA-SiO_2_-GIC	1.132 ± 0.145 ^3^	0.6 ± 0.07 ^3^	8.711 ± 1.947 ^1,2,4,5,6^	17.327 ± 1.661 ^1,2,3,5,6^	0.54 ± 0.126 ^3^	0.291 ± 0.135 ^3^
	*p*-value (*t*-test)	0.000 **	0.000 **	0.53	0.107	0.367	0.949
Aluminum	cGIC	0.204 ± 0.092 ^4^	0.226 ± 0.008 ^4^	0.805 ± 0.17 ^4^	15.3 ± 0.98 ^1,2,3,5,6^	0.176 ± 0.086 ^4^	0.165 ± 0.012 ^4^
	Nano-HA-SiO_2_-GIC	0.121 ± 0.007 ^3,4^	0.022 ± 0.007 ^3,4^	8.279 ± 1.039 ^1,2,5,6^	13.48 ± 1.662 ^1,2,5,6^	0.191 ± 0.04 ^3,4^	0.139 ± 0.044 ^3,4^
	*p*-value (*t*-test)	0.07	0.000 **	0.000 **	0.068	0.732	0.233

* Indicates significant difference between cGIC and nano-HA-SiO_2_-GIC (*p* ≤ 0.05). ** Indicates highly significant difference between cGIC and nano-HA-SiO_2_-GIC (*p* ≤ 0.05). In each row, different superscript numbers indicate significant differences between each material (*p* < 0.05).

**Table 3:** Mean and standard deviation of various selected ions in enamel and dentine at day 15 expressed in wt.%.

	Experimental Groups	Enamel			Dentine		
		0.5 mm	0.1 mm	IEL-Enamel	IEL-Dentine	0.1 mm	0.5 mm
Fluoride	cGIC	0.031 ± 0.012 ^3,4^	0.194 ± 0.008 ^3,4^	6.484 ± 1.514 ^1,2,5,6^	6.529 ± 1.952 ^1,2,5,6^	1.247 ± 0.065 ^3,4^	0.663 ± 0.023 ^3,4^
	Nano-HA-SiO_2_-GIC	0.402 ± 0.089 ^3,4^	0.644 ± 0.063 ^4^	2.076 ± 0.838 ^1,4,5,6^	9.065 ± 1.641 ^1,2,3,5,6^	0.418 ± 0.129 ^3,4^	0.118 ± 0.022 ^3,4^
	*p*-value (*t*-test)	0.000 **	0.000 **	0.000 **	0.057	0.000 **	0.000 **
Silica	cGIC	0.389 ± 0.007 ^3,4^	0.219 ± 0.012 ^3,4^	5.708 ± 2.291 ^1,2,4,5,6^	8.334 ± 1.03 ^1,2,3,5,6^	0.262 ± 0.017 ^3,4^	0.227 ± 0.016 ^3,4^
	Nano-HA-SiO_2_-GIC	0.396 ± 0.175 ^2,3,4^	3.958 ± 0.124 ^1,4,5,6^	5.324 ± 0.991 ^1,4,5,6^	11.22 ± 1.611 ^1,2,3,5,6^	0.338 ± 0.053 ^2,3,4^	0.116 ± 0.081 ^2,3,4^
	*p*-value (*t*-test)	0.934	0.000 **	0.74	0.01 *	0.015 *	0.017 *
Phosphorus	cGIC	15.529 ± 0.01 ^2,3,4,5,6^	13.046 ± 0.012 ^1,3,4,5^	5.6919 ± 1.56 ^1,2,5,6^	5.345 ± 0.472 ^1,2,5,6^	11.712 ± 0.006 ^1,2,3,4^	12.34 ± 0.011 ^1,3,4^
	Nano-HA-SiO_2_-GIC	17.173 ± 0.113 ^3,4^	16.731 ± 0.09 ^3,4^	10.841 ± 1.015 ^1,2,5,6^	7.048 ± 1.177 ^1,2,5,6^	15.744 ± 3.595 ^3,4^	16.937 ± 0.219 ^3,4^
	*p*-value (*t*-test)	0.000 **	0.000 **	0.000 **	0.017 *	0.037 *	0.000 **
Calcium	cGIC	29.792 ± 0.005 ^3,4,5,6^	25.875 ± 0.009 ^3,4^	9.9753 ± 2.434 ^1,2,4,5,6^	1.034 ± 0.007 ^1,2,3,5,6^	25.476 ± 4.036 ^1,3,4^	24.085 ± 2.328 ^1,3,4^
	Nano-HA-SiO_2_-GIC	31.675 ± 0.168 ^3,4,5^	32.377 ± 0.1 ^3,4,5^	19.517 ± 1.231 ^1,2,4,5,6^	5.161 ± 0.851 ^1,2,3,5,6^	25.719 ± 2.029 ^1,2,3,4,6^	32.39 ± 1.667 ^3,4,5^
	*p*-value (*t*-test)	0.000 **	0.000 **	0.000 **	0.000 **	0.907	0.000 **
Strontium	cGIC	0.38 ± 0.01 ^3,4^	0.451 ± 0.009 ^3,4^	8.781 ± 1.02 ^1,2,4,5,6^	14.759 ± 1.472 ^1,2,3,5,6^	1.406 ± 0.008 ^3,4^	1.033 ± 0.009 ^3,4^
	Nano-HA-SiO_2_-GIC	0.726 ± 0.251 ^3,4^	3.617 ± 0.021 ^4^	5.221 ± 1.266 ^1,4,5,6^	15.427 ± 4.109 ^1,2,3,5,6^	1.006 ± 0.062 ^3,4^	0.286 ± 0.166 ^3,4^
	*p*-value (*t*-test)	0.015 *	0.000 **	0.001 **	0.741	0.000 **	0.000 **
Aluminum	cGIC	0.009 ± 0.001 ^3,4^	0.078 ± 0.005 ^3,4^	6.633 ± 3.016 ^1,2,4,5,6^	12.14 ± 0.641 ^1,2,3,5,6^	0.2 ± 0.008 ^3,4^	0.246 ± 0.008 ^3,4^
	Nano-HA-SiO_2_-GIC	0.29 ± 0.094 ^3,4^	0.306 ± 0.048 ^3,4^	4.447 ± 1.963 ^1,2,4,5,6^	8.268 ± 3.32 ^1,2,3,5,6^	0.167 ± 0.099 ^3,4^	0.118 ± 0.01 ^3,4^
	*p*-value (*t*-test)	0.000 **	0.000 **	0.217	0.034 *	0.486	0.000 **

* Indicates significant difference between cGIC and nano-HA-SiO_2_-GIC (*p* ≤ 0.05). ** Indicates highly significant difference between cGIC and nano-HA-SiO_2_-GIC (*p* ≤ 0.05). In each row, different superscript numbers indicate significant differences between each material (*p* < 0.05).

**Table 4:** Mean and standard deviation of various selected ions in enamel and dentine at day 30 expressed in wt.%.

	Experimental Groups	Enamel			Dentine		
		0.5 mm	0.1 mm	IEL-Enamel	IEL-Dentine	0.1 mm	0.5 mm
Fluoride	cGIC	0.296 ± 0.008 ^3,4^	0.515 ± 0.126 ^3,4^	7.329 ± 1.315 ^1,2,4,5,6^	5.437 ± 1.106 ^1,2,3,5,6^	0.509 ± 0.13 ^3,4^	0.139 ± 0.01 ^3,4^
	Nano-HA-SiO_2_-GIC	0.476 ± 0.127 ^3,4^	5.262 ± 1.384 ^3,4^	5.262 ± 1.384 ^1,2,4,5,6^	8.952 ± 1.227 ^1,2,3,5,6^	1.548 ± 0.244 ^3,4^	0.199 ± 0.066 ^3,4^
	*p*-value (*t*-test)	0.13	0.083	0.042 *	0.001 **	0.000 **	0.083
Silica	cGIC	0.061 ± 0.006 ^3,4^	0.192 ± 0.068 ^3,4^	4.741 ± 1.118 ^1,2,4,5,6^	9.445 ± 1.003 ^1,2,3,5,6^	0.125 ± 0.011 ^3,4^	0.091 ± 0.013 ^3,4^
	Nano-HA-SiO_2_-GIC	0.002 ± 0.004 ^3,4,5^	0.273 ± 0.022 ^3,4,5^	8.816 ± 0.915 ^1,2,4,5,6^	6.619 ± 0.774 ^1,2,3,5,6^	2.622 ± 0.216 ^1,2,3,4,6^	0.076 ± 0.011 ^3,4,5^
	*p*-value (*t*-test)	0.000 **	0.036 *	0.000 **	0.001 **	0.000 **	0.089
Phosphorus	cGIC	13.348 ± 0.007 ^3,4,5,6^	13.753 ± 0.108 ^3,4,5,6^	3.2137 ± 1.341 ^1,2,5,6^	4.13 ± 0.793 ^1,2,5,6^	11.707 ± 0.157 ^1,2,3,4^	11.858 ± 0.006 ^1,2,3,4^
	Nano-HA-SiO_2_-GIC	16.549 ± 0.121 ^3,4^	16.322 ± 0.405^3,4^	7.5666 ± 1.404 ^1,2,5,6^	5.659 ± 0.579 ^1,2,5,6^	14.988 ± 0.785 ^3,4^	16.561 ± 1.325 ^3,4^
	*p*-value (*t*-test)	0.000 **	0.000 **	0.001 **	0.008 **	0.000 **	0.000 **
Calcium	cGIC	26.62 ± 0.015 ^3,4^	27.63 ± 0.065 ^3,4^	5.2985 ± 1.154 ^1,2,5,6^	2.985 ± 0.072 ^1,2,5,6^	23.522 ± 4.752 ^3,4^	24.418 ± 3.346 ^3,4^
	Nano-HA-SiO_2_-GIC	30.861 ± 0.16 ^3,4,5^	29.777 ± 1.016 ^3,4,5^	11.832 ± 0.972 ^1,2,5,6^	11.22 ± 1.378 ^1,2,5,6^	21.667 ± 0.506 ^1,2,3,4,6^	30.417 ± 4.497 ^3,4,5^
	*p*-value (*t*-test)	0.000 **	0.002 **	0.000 **	0.000 **	0.411	0.044 *
Strontium	cGIC	0.171 ± 0.015 ^3,4^	0.619 ± 0.136 ^3,4^	7.474 ± 0.826 ^1,2,4,5,6^	11.688 ± 0.876 ^1,2,3,5,6^	0.643 ± 0.113 ^3,4^	0.002 ± 0.004 ^3,4^
	Nano-HA-SiO_2_-GIC	1.365 ± 0.092 ^3,4^	1.155 ± 0.397 ^3,4^	9.603 ± 2.056 ^1,2,4,5,6^	15.085 ± 1.832 ^1,2,3,5,6^	1.766 ± 0.739 ^3,4^	0.874 ± 0.065 ^3,4^
	*p*-value (*t*-test)	0.000 **	0.021 *	0.064	0.006 **	0.01 *	0.000 **
Aluminum	cGIC	0.15 ± 0.008 ^3,4^	0.075 ± 0.015 ^3,4^	6.787 ± 1.307 ^1,2,4,5,6^	11.15 ± 1.054 ^1,2,3,5,6^	0.146 ± 0.021 ^3,4^	0.053 ± 0.008 ^3,4^
	Nano-HA-SiO_2_-GIC	0.161 ± 0.084 ^3,4^	0.212 ± 0.069 ^3,4^	7.61 ± 0.687 ^1,2,4,5,6^	5.066 ± 1.284 ^1,2,3,5,6^	1.234 ± 0.025 ^3,4^	0.039 ± 0.004 ^3,4^
	*p*-value (*t*-test)	0.768	0.002 **	0.248	0.000 **	0.000 **	0.007 **

* Indicates significant difference between cGIC and nano-HA-SiO_2_-GIC (*p* ≤ 0.05). ** Indicates highly significant difference between cGIC and nano-HA-SiO_2_-GIC (*p* ≤ 0.05). In each row, different superscript numbers indicate significant differences between each material (*p* < 0.05).

## Data Availability

Not applicable.

## Supplementary Materials

The following are available online at https://www.mdpi.com/article/10.3390/polym13203504/s1, Table S1: Ion-concentration of various selected elements in enamel and cGIC at various time intervals expressed in wt%, Table S2: Ion-concentration of various selected elements in dentine and cGIC at various time intervals expressed in wt%, Table S3: Ion-concentration of various selected elements in enamel and nano-HA-SiO2-GIC at various time intervals expressed in wt%, Table S4: Ion-concentration of various selected elements in enamel and nano-HA-SiO2-GIC at various time intervals expressed in wt%.
