# Supplementary material for: Analysis of Ionic-Exchange of Selected Elements between Novel Nano-Hydroxyapatite-Silica Added Glass Ionomer Cement and Natural Teeth
Source: Polymers (Basel). 2021 Oct 12;13(20):3504. doi: 10.3390/polym13203504 (PMC8537145; doi:10.3390/polym13203504)
Supplement: Supplementary file 1 [file polymers-13-03504-s001.zip › polymers-1388730-supplementary.pdf]

Table S1. Ion-concentration of various selected elements in enamel and cGIC at various time intervals expressed in wt%.

	Natural Enamel	Enamel		IEL	cGIC	
		.5 mm	.1 mm		.1 mm	.5 mm
**F – Day 3**	0 ± 0	0.77 ± 0.1	0.13 ± 0.05	6.96 ± 0.68	12.5 ± 1.37	9.18 ± 0.51
**F – Day 7**	0 ± 0	1.1 ± 0.15	0.92 ± 0.34	4.68 ± 1.53	6.02 ± 0.72	13.4± 1.6
**F – Day 15**	0 ± 0	0.03 ± 0.0	0.19 ± 0.01	6.48 ± 1.51	7.41 ± 0.51	6.56 ± 1.68
**F – Day 30**	0 ± 0	0.3 ± 0.01	0.51 ± 0.13	7.33 ± 1.32	4.57 ± 0.95	6.59 ± 1.11
**Si – Day 3**	0.27 ± 0.03	0 ± 0	0.14 ± 0.05	6.37 ± 0.68	12.3 ± 0.8	15.1 ± 0.94
**Si – Day 7**	0.27 ± 0.03	0.08 ± 0.01	0.63 ± 0.21	5.84 ± 1.75	12.3 ± 2.06	11 ± 0.9
**Si – Day 15**	0.27 ± 0.03	0.39 ± 0.01	0.22 ± 0.01	5.71 ± 2.29	8.9 ± 1.48	6.14 ± 0.79
**Si – Day 30**	0.27 ± 0.03	0.06 ± 0.01	0.19 ± 0.07	4.74 ± 1.12	7.01 ± 0.87	7.69 ± 1.05
**P – Day 3**	17.6 ± 1.16	17.8 ± 0.13	17.5 ± 0.03	16.4 ± 0.28	3.35 ± 0.75	2.68 ± 0.88
**P – Day 7**	17.6 ± 1.16	17.6 ± 0.15	17.9 ± 0.47	14.1 ± 1.5	3.44 ± 1.69	1.33 ± 0.79
**P – Day 15**	17.6 ± 1.16	15.5 ± 0.01	13 ± 0.01	5.69 ± 1.56	1.96 ± 0.8	3.5 ± 1.26
**P – Day 30**	17.6 ± 1.16	13.3 ± 0.01	13.8 ± 0.11	3.21 ± 1.34	4.81 ± 0.93	1.26 ± 0.43
**Ca – Day 3**	32.9 ± 2.37	37.4 ± 0.19	36.3 ± 0.02	36.2 ± 0.56	0.61 ± 0.15	1.48 ± 0.25
**Ca – Day 7**	32.9 ± 2.37	37.5 ± 0.27	33.8 ± 0.24	31.4 ± 1.19	0.93 ± 0.11	0.85 ± 0.33
**Ca – Day 15**	32.9 ± 2.37	29.8 ± 0	25.9 ± 0.01	9.98 ± 2.43	1.04 ± 0.08	1.68 ± 0.38
**Ca – Day 30**	32.9 ± 2.37	26.6 ± 0.02	27.6 ± 0.07	5.3 ± 1.15	4.14 ± 1.51	0.85 ± 0.19
**Sr – Day 3**	0 ± 0	0 ± 0	0.7± 0.02	6.4 ± 0.39	21.6 ± 1.25	22.4 ± 0.48
**Sr – Day 7**	0 ± 0	0.42 ± 0.04	0.27 ± 0.1	6.02 ± 1.8	25.6 ± 2.27	18.5 ± 0.87
**Sr – Day 15**	0 ± 0	0.38 ± 0.01	0.45 ± 0.01	8.78 ± 1.02	7.81 ± 0.83	16.1 ± 1.9
**Sr – Day 30**	0 ± 0	0.17 ± 0.01	0.62 ± 0.14	7.47 ± 0.83	9.47 ± 1.21	16 ± 1.5
**Al – Day 3**	0 ± 0	0.09 ± 0.01	0 ± 0	1 ± 0.35	13.2 ± 0.59	15 ± 0.88
**Al – Day 7**	0 ± 0	0.2 ± 0.09	0.23 ± 0.01	0.81 ± 0.17	12.1 ± 1.1	13.6 ± 1.18
**Al – Day 15**	0 ± 0	0.01 ± 0	0.08 ± 0.01	6.63 ± 3.02	8.88 ± 3.43	7.8 ± 1.39
**Al – Day 30**	0 ± 0	0.15 ± 0.01	0.08 ± 0.01	6.79 ± 1.31	7.64 ± 1.13	8.93 ± 0.39

Table S2. Ion-concentration of various selected elements in dentine and cGIC at various time intervals expressed in wt%.

	Natural dentine	Dentine		IEL	cGIC	
		.5 mm	.1 mm		.1 mm	.5 mm
**F – Day 3**	0 ±0	0.49 ± 0.07	0.11 ± 0.04	8.7 ± 0.69	9.03 ± 1.48	13.4 ± 0.35
**F – Day 7**	0 ±0	0.94 ± 0.18	3.03 ± 0.3	10.1 ± 1.25	9.99 ± 1.6	10.6 ± 1.81
**F – Day 15**	0 ±0	0.66 ± 0.02	1.25 ± 0.07	6.53 ± 1.95	5.44 ± 2.93	6.7 ± 0.77
**F – Day 30**	0 ±0	0.14 ± 0.01	0.51 ± 0.13	5.44 ± 1.11	2.76 ± 1.23	7.78 ± 1.66
**Si – Day 3**	0 ± 0.01	0.16 ± 0.04	6.99 ± 0.6	12.2 ± 0.6	14.4 ± 0.7	0.25 ± 0.08
**Si – Day 7**	0.25 ± 0.08	0.07 ± 0.01	0.45 ± 0.22	12.5 ± 1.55	13.8 ± 0.64	14.8 ± 0.82
**Si – Day 15**	0.25 ± 0.08	0.23 ± 0.02	0.26 ± 0.02	8.33 ± 1.03	8.77 ± 2.9	7.37 ± 1.29
**Si – Day 30**	0.25 ± 0.08	0.09 ± 0.01	0.12 ± 0.01	9.45 ± 1	9.74 ± 1.16	7.35 ± 1.17
**P – Day 3**	14.6 ± 0.79	17.9 ± 0.16	17.5 ± 0.04	2.68 ± 0.4	1.6 ± 0.48	3.2 ± 0.63
**P – Day 7**	14.6 ± 0.79	17.5 ± 0.18	17.8 ± 0.2	3.45 ± 2.22	2.57 ± 0.95	4.02 ± 0.82
**P – Day 15**	14.6 ± 0.79	12.3 ± 0.01	11.7 ± 0.01	5.34 ± 0.47	7.21 ± 2.04	3.3 ± 0.9
**P – Day 30**	14.6 ± 0.79	11.9 ± 0.01	11.7 ± 0.16	4.13 ± 0.79	5.16 ± 0.78	3.23 ±0.81
**Ca – Day 3**	25.8 ± 4.69	36.4 ± 2.42	37.1 ± 4.14	0.65 ± 0.45	2.7 ± 0.57	0.61 ± 0.03
**Ca – Day 7**	25.8 ± 4.69	36.9 ± 2.61	33.9 ± 1.63	0.14 ± 0.01	0.56 ± 0.03	0.58 ± 0.08
**Ca – Day 15**	25.8 ± 4.69	24.1 ± 2.33	25.5 ± 4.04	1.03 ± 0.01	12.9 ± 1.6	2.55 ± 0.92
**Ca – Day 30**	25.8 ± 4.69	24.4 ± 3.35	23.5 ± 4.75	2.98 ± 0.07	6.75 ± 1.77	1.34 ±0.5
**Sr – Day 3**	0 ±0	0 ±0	0.68 ± 0.05	19.7 ± 1.52	18.1 ± 0.89	18.2 ± 0.37
**Sr – Day 7**	0 ±0	0.3 ± 0.15	0.62 ± 0.15	20.2 ± 3.13	19.9 ± 1.12	23.2 ± 1.08
**Sr – Day 15**	0 ±0	1.03 ± 0.01	1.41 ± 0.01	14.8 ± 1.47	17.1 ± 1.64	16.4 ± 1.59
**Sr – Day 30**	0 ±0	0 ±0	0.64 ± 0.11	11.7 ± 0.88	16.6 ± 1.95	16.2 ± 1.94
**Al – Day 3**	0 ±0	0.1 ± 0	0 ±0	11.9 ± 2.02	14.9 ± 0.7	12.2 ± 1.26
**Al – Day 7**	0 ±0	0.16 ± 0.01	0.18 ± 0.09	15.3 ± 0.98	15.1 ± 1.24	15.2 ± 1.85
**Al – Day 15**	0 ±0	0.25 ± 0.01	0.2 ± 0.01	12.1 ± 0.64	11.1 ± 2.64	9.3 ± 1.1
**Al – Day 30**	0 ±0	0.05 ± 0.01	0.15 ± 0.02	11.1 ± 1.05	10.4 ± 1.14	10.1 ± 1.49

Table S3. Ion-concentration of various selected elements in enamel and nano-HA-SiO_2-GIC at various time intervals expressed in wt%.

	**Natural enamel**	**Enamel .5 mm**	**Enamel .1 mm**	**IEL**	**nano-HA-SiO$_2$-GIC .1 mm**	**nano-HA-SiO$_2$-GIC .5 mm**
**F – Day 3**	0 ±0	1.03 ± 0.31	0.39 ± 0.11	2.62 ± 0.03	6.42 ± 1.5	15.52 ± 1.37
**F – Day 7**	0 ± 0	0.68 ± 0.17	0.63 ± 0.09	4.12 ± 2.39	7.80 ± 1.14	13.08 ± 1.32
**F – Day 15**	0 ± 0	0.40 ± 0.09	0.64 ± 0.06	2.08 ± 0.84	10.22 ± 1.64	14.33 ± 1.31
**F – Day 30**	0 ± 0	0.48 ± 0.13	0.40 ± 0.01	5.26 ± 1.38	13.74 ± 0.96	9.82 ± 0.6
**Si – Day 3**	0.27 ± 0.03	0.22 ± 0.26	0.00 ± 0	11.33 ± 0.04	6.23 ± 1.5	14.52 ± 1.22
**Si – Day 7**	0.27 ± 0.03	0.69 ± 0.18	0.27 ± 0.07	11.95 ± 1.54	7.83 ± 0.93	15.4 ± 1.14
**Si – Day 15**	0.27 ± 0.03	0.340 ± 0.18	3.96 ± 0.12	5.324 ± 0.99	9.50 ± 1.92	16.41 ± 1.03
**Si – Day 30**	0.27 ± 0.03	0.002 ± 0	0.27 ± 0.02	8.82 ± 0.92	7.35 ± 1.91	13.69 ± 2.16
**P – Day 3**	17.6 ± 1.16	16.88 ± 0.29	16.95 ± 0.06	17.06 ± 0.02	6.98 ± 0.69	5.55 ± 0.91
**P – Day 7**	17.6 ± 1.16	15.98 ± 0.15	17.41 ± 0.08	6.35 ± 1.34	5.11 ± 1.46	5.46 ± 0.87
**P – Day 15**	17.6 ±1.16	17.17 ± 0.11	16.73 ± 0.09	10.84 ± 1.01	4.85 ± 1.13	2.68 ± 0.72
**P – Day 30**	17.6 ± 1.16	16.55 ± 0.12	16.32 ± 0.41	7.57 ± 1.4	4.07 ± 0.35	4.03 ± 0.79
**Ca – Day 3**	32.93 ± 2.37	32.97 ± 0.13	35.56 ± 0.07	24.78 ± 0.04	19.64 ± 0.83	12.54 ± 0.86
**Ca – Day 7**	32.93 ± 2.37	29.71 ± 0.12	34.02 ± 0.05	11.28 ± 3.07	11.29 ± 1.73	11.16 ± 1.65
**Ca – Day 15**	32.93 ± 2.37	31.67 ± 0.17	32.38 ± 0.1	19.52 ± 1.23	8.56 ± 1.11	3.11 ± 0.93
**Ca – Day 30**	32.93 ± 2.37	30.86 ± 0.16	29.78 ± 1.02	11.83 ± 0.97	4.92 ± 1.73	1.01 ± 0.68
**Sr – Day 3**	0 ± 0	1.19 ± 0.23	0.13 ± 0.06	7.41 ± 0.02	8.45 ± 1.45	18.96 ± 1.4
**Sr – Day 7**	0 ± 0	1.13 ± 0.15	0.6 ± 0.07	8.71 ± 1.95	6.41 ± 0.89	21.91 ± 1.42
**Sr – Day 15**	0 ± 0	0.73 ± 0.25	3.62 ± 0.02	5.22 ± 1.27	12.6 ± 1.36	15.27 ± 1.54
**Sr – Day 30**	0 ± 0	1.37 ± 0.09	1.16 ± 0.4	9.60 ± 2.06	14.29 ± 1.18	21.12 ± 1.42
**Al – Day 3**	0 ± 0	0.14 ± 0.07	0.19 ± 0.12	7.07 ± 0.02	8.38 ± 0.31	15.31 ± 1.15
**Al – Day 7**	0 ± 0	0.12 ± 0.01	0.02 ± 0.01	8.28 ± 1.04	6.62 ± 1.88	14.43 ± 1.27
**Al – Day 15**	0 ± 0	0.29 ± 0.09	0.31 ± 0.05	4.45 ± 1.96	8.84 ± 1.1	11.78 ± 1.73
**Al – Day 30**	0 ± 0	0.16 ± 0.08	0.21 ± 0.07	7.61 ± 0.69	10.27 ± 1.3	15.39 ± 1.52

Table S4. Ion-concentration of various selected elements in enamel and nano-HA-SiO_2-GIC at various time intervals expressed in wt%.

	Natural dentine	Dentine		IEL	nano-HA-SiO_2-GIC	
		.5 mm	.1 mm		.1 mm	.5 mm
**F – Day 3**	0 ± 0	0.1 ± 0.01	0.05 ± 0.02	6.84 ± 0.02	9.9 ± 1.59	8.99 ± 0.88
**F – Day 7**	0 ± 0	0.08 ± 0.04	1.2 ± 0.06	4.95 ± 1.07	12.3 ± 0.86	11.9 ± 0.07
**F – Day 15**	0 ± 0	0.12 ± 0.02	0.42 ± 0.13	9.07 ± 1.64	8.21 ± 1.34	9.08 ± 0.26
**F – Day 30**	0 ± 0	0.2 ± 0.07	1.55 ± 0.24	8.95 ± 1.23	13.9 ± 1.44	10.3 ± 1.07
**Si – Day 3**	0.25 ± 0.08	0.24 ± 0.05	0.56 ± 0.17	8.97 ± 0.26	7.89 ± 0.72	10.5 ± 0.78
**Si – Day 7**	0.25 ± 0.08	0.3 ± 0.05	0.31 ± 0.08	17.5 ± 1.01	13.6 ± 1.64	16.2 ± 1.19
**Si – Day 15**	0.25 ± 0.08	0.12 ± 0.08	0.34 ± 0.05	11.2 ± 1.61	10.1 ± 1.76	11.9 ± 1.11
**Si – Day 30**	0.25 ± 0.08	0.08 ± 0.01	2.62 ± 0.22	6.62 ± 0.77	11.7 ± 1.54	12 ± 1.5
**P – Day 3**	14.6 ± 0.79	15.9 ± 1.76	15.3 ± 3.89	6.43 ± 0.02	10 ± 0.63	6.29 ± 1.42
**P – Day 7**	14.6 ± 0.79	16.6 ± 1.82	16.9 ± 1.24	4.58 ± 1.49	2.53 ± 0.96	2.65 ± 0.75
**P – Day 15**	14.6 ± 0.79	16.9 ± 0.22	15.7 ± 3.6	7.05 ± 1.18	5.13 ± 1.04	5.58 ± 0.92
**P – Day 30**	14.6 ± 0.79	16.6 ± 1.33	15 ± 0.78	5.66 ± 0.58	2.75 ± 1.17	7.21 ± 2.13
**Ca – Day 3**	25.8 ± 4.69	32 ± 2.88	30.7 ± 1.6	8.27 ± 0.03	16.8 ± 1.76	9.06 ± 0.61
**Ca – Day 7**	25.8 ± 4.69	30.9 ± 0.85	31 ± 2.52	11.2 ± 1.23	10.7 ± 0.86	6.34 ± 0.41
**Ca – Day 15**	25.8 ± 4.69	32.4 ± 1.67	25.7 ± 2.03	5.16 ± 0.85	8.02 ± 1.51	8.02 ± 1.08
**Ca – Day 30**	25.8 ± 4.69	30.4 ± 4.5	21.7 ± 0.51	11.2 ± 1.38	12.6 ± 1.75	9.31 ± 1.24
**Sr – Day 3**	0 ± 0	1.12 ± 0.18	1.56 ± 0.08	12.8 ± 1.01	11.8 ± 1.21	12.1 ± 0.51
**Sr – Day 7**	0 ± 0	0.29 ± 0.14	0.54 ± 0.13	17.3 ± 1.66	14.9 ± 1.41	18 ± 1.95
**Sr – Day 15**	0 ± 0	0.29 ± 0.17	1.01 ± 0.06	15.4 ± 4.11	14.7 ± 0.77	12.9 ± 1.74
**Sr – Day 30**	0 ± 0	0.87 ± 0.06	1.77 ± 0.74	15.1 ± 1.83	15.4 ± 2.07	13.2 ± 2.36
**Al – Day 3**	0 ± 0	0.39 ± 0.1	0.24 ± 0.11	8.56 ± 0.03	7.3 ± 0.59	10.4 ± 1.1
**Al – Day 7**	0 ± 0	0.14 ± 0.04	0.19 ± 0.04	13.5 ± 1.66	11.6 ± 1.95	13.7 ± 1.86
**Al – Day 15**	0 ± 0	0.12 ± 0.01	0.17 ± 0.1	8.27 ± 3.32	12.4 ± 1.06	9.56 ± 1.21
**Al – Day 30**	0 ± 0	0.04 ± 0	1.23 ± 0.02	5.07 ± 1.28	11.7 ± 0.86	9.51 ± 1.56
